# Intramuscular injection of mesenchymal stem cells augments basal muscle protein synthesis after bouts of resistance exercise in male mice

**DOI:** 10.14814/phy2.15991

**Published:** 2024-04-11

**Authors:** Junya Takegaki, Kohei Sase, Yusuke Kono, Takuya Fujita, Satoshi Konishi, Satoshi Fujita

**Affiliations:** ^1^ Research Organization of Science and Technology Ritsumeikan University Kusatsu Shiga Japan; ^2^ Ritsumeikan Global Innovation Research Organization Ritsumeikan University Kusatsu Shiga Japan; ^3^ Graduate School of Agricultural Science Kobe University Kobe Hyogo Japan; ^4^ Faculty of Sport and Health Science Ritsumeikan University Kusatsu Shiga Japan; ^5^ Faculty of Pharmaceutical Sciences Kobe Pharmaceutical University Kobe Hyogo Japan; ^6^ College of Pharmaceutical Sciences Ritsumeikan University Kusatsu Shiga Japan; ^7^ Faculty of Science and Engineering Ritsumeikan University Kusatsu Shiga Japan

**Keywords:** insulin‐like growth factor 1, mesenchymal stem cells, muscle protein synthesis, protein ubiquitination, resistance exercise

## Abstract

Skeletal muscle mass is critical for activities of daily living. Resistance training maintains or increases muscle mass, and various strategies maximize the training adaptation. Mesenchymal stem cells (MSCs) are multipotent cells with differential potency in skeletal muscle cells and the capacity to secrete growth factors. However, little is known regarding the effect of intramuscular injection of MSCs on basal muscle protein synthesis and catabolic systems after resistance training. Here, we measured changes in basal muscle protein synthesis, the ubiquitin‐proteasome system, and autophagy‐lysosome system‐related factors after bouts of resistance exercise by intramuscular injection of MSCs. Mice performed three bouts of resistance exercise (each consisting of 50 maximal isometric contractions elicited by electrical stimulation) on the right gastrocnemius muscle every 48 h, and immediately after the first bout, mice were intramuscularly injected with either MSCs (2.0 × 10^6^ cells) labeled with green fluorescence protein (GFP) or vehicle only placebo. Seventy‐two hours after the third exercise bout, GFP was detected only in the muscle injected with MSCs with concomitant elevation of muscle protein synthesis. The injection of MSCs also increased protein ubiquitination. These results suggest that the intramuscular injection of MSCs augmented muscle protein turnover at the basal state after consecutive resistance exercise.

## 1 INTRODUCTION

Skeletal muscle regulates body movement, and muscle mass is critical for daily activities. Resistance training increases skeletal muscle mass and is widely used as an effective countermeasure against skeletal muscle atrophy (e.g., aging and bedrest) (Alkner & Tesch, 2004; Talar et al., 2021). There are growing number of additional interventions to accelerate the effect of resistance training. Nutritional interventions such as whey protein or essential amino acid ingestions are used, but some kinds of atrophic conditions, including sarcopenia, induce anabolic resistance (Haran et al., 2012). Therefore, a new modality to complement resistance training adaptations is needed.

The net balance between muscle protein synthesis and muscle protein breakdown maintains skeletal muscle mass. Resistance exercise effectively activates muscle protein synthesis, and repeated resistance exercise bouts induce skeletal muscle hypertrophy (Brook et al., 2015; Phillips et al., 1997). Both in humans and rodents, two to three bouts of exercise per week can evoke skeletal muscle hypertrophy (Brook et al., 2015; Ogasawara et al., 2016; Takegaki et al., 2019). The transient activation of muscle protein synthesis by a single bout of resistance exercise peaks at the early phase after the exercise (within a few hours post‐exercise) and returns to a basal level within 24–48 h (Ogasawara et al., 2016; Phillips et al., 1997; West et al., 2016). Although nutritional interventions, including protein or amino acids intake, are widely utilized to augment the transient activation of muscle protein synthesis, the effect on basal muscle protein synthesis remains understudied (Bukhari et al., 2015; Churchward‐Venne et al., 2012; Tang et al., 2009; Yang et al., 2012). A decrease in basal muscle protein synthesis (in immobilization or reduced physical activity) evokes skeletal muscle atrophy (Brook et al., 2022; McGlory et al., 2018). On the other hand, a previous study reported that an increase in basal muscle protein synthesis by long‐term nutritional supplementation coincides with an increase in lean body mass (Dillon et al., 2009). These facts suggest that increasing the basal state of muscle protein synthesis may further facilitate resistance exercise‐induced increase in muscle mass. However, little is available on achieving chronic activation of muscle protein synthesis or an increase in the basal state of protein synthesis in response to resistance training.

Mesenchymal stem cells (MSCs) are multipotent stem cells that differentiate into mesenchymal cells, including muscle cells (Dezawa et al., 2005). In skeletal muscle, MSCs promote the regeneration process after muscle injury (Linard et al., 2018; Ninagawa et al., 2013; Oshima et al., 2014; Winkler et al., 2008). Also, MSCs secrete growth factors, including insulin‐like growth factor‐1 (IGF‐1), that stimulate muscle protein synthesis (Crisostomo et al., 2008; Rommel et al., 2001). Studies reported positive effects of MSCs on muscle mass in mice (e.g., downhill running‐induced muscle hypertrophy and high‐fat diet‐induced muscle atrophy) (Abrigo et al., 2016; Zou et al., 2015). Recently, we showed that intramuscularly injected MSCs remained in the muscle for 7 days and increased basal muscle protein synthesis in mice (Takegaki et al., 2021). Accordingly, MSCs may help augment muscle protein synthesis at the basal state after resistance training from these observations.

In the present study, we investigated the effect of intramuscular injection of MSCs on muscle protein synthesis, protein catabolic systems, and factors related to muscle satellite cells and IGF‐1 in the basal state after three bouts of resistance exercise in mouse skeletal muscle. We hypothesized that intramuscular injection of MSCs would augment muscle protein synthesis at the basal state after bouts of resistance exercise.

## 2 METHODS

### 2.1 Animals and experimental design

**FIGURE 1:**
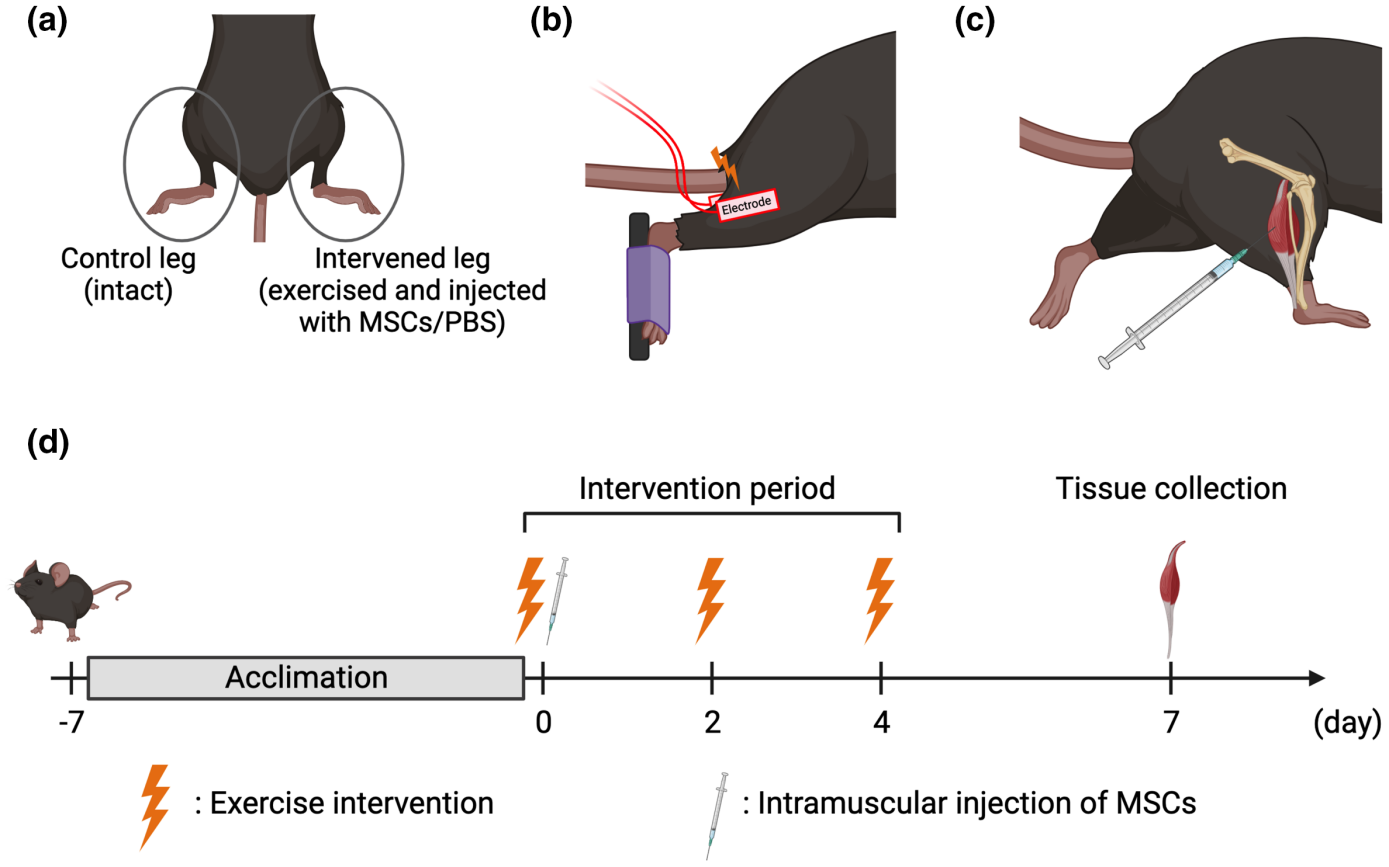
Interventions and experimental design. Use of each leg (a), electrical stimulation on gastrocnemius muscle (b), intramuscular injection (c), and experimental design (d). The left leg was used as intact control, and the right leg was used as intervention. Intervened legs were exercised and injected with mesenchymal stem cells (MSCs)/phosphate‐buffered saline (PBS). Resistance exercise was performed on the gastrocnemius muscle by transcutaneous electrical stimulation under anesthesia. The intramuscular injection of MSCs/PBS was performed after the first bout of resistance exercise. Bouts of the resistance exercise were performed every other day, and gastrocnemius muscle was collected 72 h after the third exercise session.

Twelve 10‐week‐old male C57BL/6J mice were obtained from Shimizu Laboratory Supplies (Kyoto, Japan). All animals were housed at 23 ± 1°C under a 12‐h/12‐h light–dark cycle and provided food (MF; Oriental Yeast Co., Ltd., Tokyo, Japan) and water ad libitum. Mice were divided into MSC and phosphate‐buffered saline (PBS) groups. All mice performed three bouts of resistance exercise on their right gastrocnemius muscle with 48‐h recovery periods between bouts. Immediately after the first exercise bout, MSCs or vehicle PBS were injected into the exercised muscle. The left gastrocnemius muscle remained intact and was used as the internal control. Seventy‐two hours after the third resistance exercise bout, mice were anesthetized and euthanatized by cervical dislocation, and muscle samples were collected (Figure 1a–d). Three hours before the sample collection, the mice were fasted. Muscles were frozen at −80°C until use. The Ethics Committee for Animal Experiments at Ritsumeikan University approved all experiments (BKC2018‐046).

### 2.2 Resistance exercise protocol

Resistance exercise was carried out according to a protocol described previously (Ogasawara et al., 2014; Takegaki et al., 2020). Briefly, under 2% isoflurane inhalation anesthesia, the hair on the right hind limb of each mouse was removed, and the skin was cleaned with alcohol wipes. The right ankle joint was fixed at 90° relative to the tibia in the prone position. Electrodes (Vitrode V, Ag/AgCl; Nihon Kohden, Tokyo, Japan) were placed on the gastrocnemius muscle, and the muscle was stimulated percutaneously to evoke maximal isometric contractions. A bout of resistance exercise was performed by five sets of 3 s ×10 contractions with a seven‐second interval between contractions and 3‐min rest intervals between sets.

### 2.3 Cell culture and intramuscular injection

C57BL/6 mouse bone marrow‐derived MSCs with green fluorescence protein (GFP; from KAC Co., Ltd., Kyoto, Japan) were cultured in Mouse Mesenchymal Stem Cell Culture Medium (MUXMX‐90011, Cyagen Biosciences Inc., Santa Clara, CA) at 37°C in 5% CO_2_. Mice in the MSC group were intramuscularly injected with 2.0 × 10^6^ MSCs into the right gastrocnemius muscle. Mice in the PBS group were injected with PBS only. The cells used in this study are identical to those used in previous studies (Kono et al., 2021; Takegaki et al., 2021).

### 2.4 Muscle protein synthesis

Muscle protein synthesis was measured by the SUnSET method (Goodman et al., 2011). Under isoflurane anesthesia, 0.04 μmol/g bodyweight puromycin (164‐23154, FUJIFILM Wako Pure Chemical Co., Tokyo, Japan) diluted in PBS was intraperitoneally injected into each mouse. Fifteen minutes after the puromycin injection, the gastrocnemius muscles were removed. Following homogenization, samples were centrifuged at 2000 × *g* for 3 min at 4°C, and the supernatants were collected and processed for western blotting using an anti‐puromycin antibody (Cat# MABE343, Merck Millipore).

### 2.5 Western blotting

Muscle samples were homogenized and analyzed as previously described with slight modifications (Takegaki et al., 2021). Briefly, samples were homogenized in RIPA buffer (Cell Signaling Technology, Danvers, MA, USA) containing cOmplete Mini protease inhibitor cocktail and PhosSTOP phosphatase inhibitor cocktail (11836153001 and 4906845001, respectively, Sigma‐Aldrich, St. Louis, MO, USA) and centrifuged at 10,000 × *g* for 10 min at 4°C. After determination of the protein concentration of the supernatant using a Protein Assay Rapid Kit Wako II (295–78,401, FUJIFILM Wako Pure Chemical, Osaka, Japan), samples were diluted in 3× Blue Loading Buffer (#7722, Cell Signaling Technology) and boiled at 95°C for 5 min. Equal proteins were then separated on m‐PAGEL (2332240, ATTO, Tokyo, Japan) and transferred to polyvinylidene difluoride membranes. After 5 min blocking with Bullet Blocking One for Western Blotting (13779‐01, Nacalai Tesque, Kyoto, Japan) at room temperature, the membranes were incubated overnight at 4°C with following primary antibodies: GFP (#2956, Cell Signaling Technology), platelet‐derived growth factor receptor alpha (PDGFRα, #3174, Cell Signaling Technology), phosphor‐Akt (Ser473, #9275, Cell Signaling Technology), total‐Akt (#4691, Cell Signaling Technology), phosphor‐p70S6K (Thr389, #9234, Cell Signaling Technology), total‐p70S6K (#2708, Cell Signaling Technology), phosphor‐rpS6 (Ser240/244, #2215, Cell Signaling Technology), total‐rpS6 (#2217, Cell Signaling Technology), phosphor‐4EBP1 (Thr37/46, #9459, Cell Signaling Technology), total‐4EBP1 (#9644, Cell Signaling Technology), ubiquitinated proteins (#3936, Cell Signaling Technology), K48‐linkage‐specific polyubiquitin (#8081, Cell Signaling Technology), Atrogin‐1 (ab168372, Abcam, Cambridge, UK), MuRF‐1 (sc‐398608, Santa Cruz Biotechnology, Dallas, TX, USA), LC3 (#2775, Cell Signaling Technology), p62/SQSTM (PM045, Medical & Biological Laboratory, Nagoya, Japan), phosphor‐ULK1 (Ser757, #14202, Cell Signaling Technology; Ser555, #5869, Cell Signaling Technology), total‐ULK1 (#8054, Cell Signaling Technology), IGFBP3 (10189‐2‐AP, Proteintech, Rosemont, IL, USA), or IGFBP5 (55205‐1‐AP, Proteintech). After washing, membranes were incubated with the appropriate secondary antibodies (#7074 or #7076, Cell Signaling Technology) for 1 h at room temperature. Bands were visualized using Immobilon Forte Western HRP Substrate (WBLUF0500, Millipore) and detected using the FUSION Chemiluminescence Imaging System (M&S Instruments, Osaka, Japan). Band intensities were quantified using Image J software (National Institutes of Health, Bethesda, MD, USA). Ponceau S staining was used to verify equal loading between lanes and normalization. Phosphorylated proteins were also normalized to the number of proteins detected by ponceau S staining, as the intervention may change some individual total protein expressions.

### 2.6 RNA extraction and real‐time qPCR

Total RNA was extracted from each sample using TRIzol reagent (15596018, Invitrogen, Carlsbad, CA, USA), and the concentrations of RNA were measured using a NanoDrop 2000 (Thermo Fisher Scientific, Waltham, MA, USA). Using a high‐capacity cDNA RT kit (4368814, Applied Biosystems, Foster City, CA, USA), 1.5 μg of total RNA was reverse‐transcribed into cDNA. Gene expression levels were quantified using THUNDERBIRD SYBR qPCR Mix (QPS‐201, Toyobo, Osaka, Japan) with a 7500 Fast Sequence Detection System (Applied Biosystems). The gene expressions were quantified using the calibration curve method. Based on the housekeeping gene expression data in each group's pool sample (Figure S1), we used *18s* as the control housekeeping gene. Table 1 shows the primer sequences.

**TABLE 1:** Primer sequences for qPCR.

Gene	Forward primer (5′‐3′)	Reverse primer (5′‐3′)
*Pax7*	GTGCCCTCAGTGAGTTCGATTAGC	CCACATCTGAGCCCTCATCCA
*MyoD*	TGGCATGATGGATTACAGCG	GAGATGCGCTCCACTATGCT
*Myogenin*	TCCCAACCCAGGAGATCATT	TCAGTTGGGCATGGTTTCGT
*Igf‐1*	CAAGCCCACAGGCTATGGC	TCTGAGTCTTGGGCATGTCAG
*18s*	CCTGGATACCGCAGCTAGGA	GCGGCGCAATACGAATGCCCC

### 2.7 Statistical analysis

For a comparison of muscle protein synthesis relative to the internal control leg, a *t*‐test was used. Two‐way ANOVA (MSCs × exercise) was used to compare the other items. If an interaction was observed, Holm‐Šídák multiple‐comparison testing was performed. All values were expressed as mean ± SD with individual plots. The statistical significance of differences was defined as *p* < 0.05.

## 3 RESULTS

### 3.1 Markers of MSCs

**FIGURE 2:**
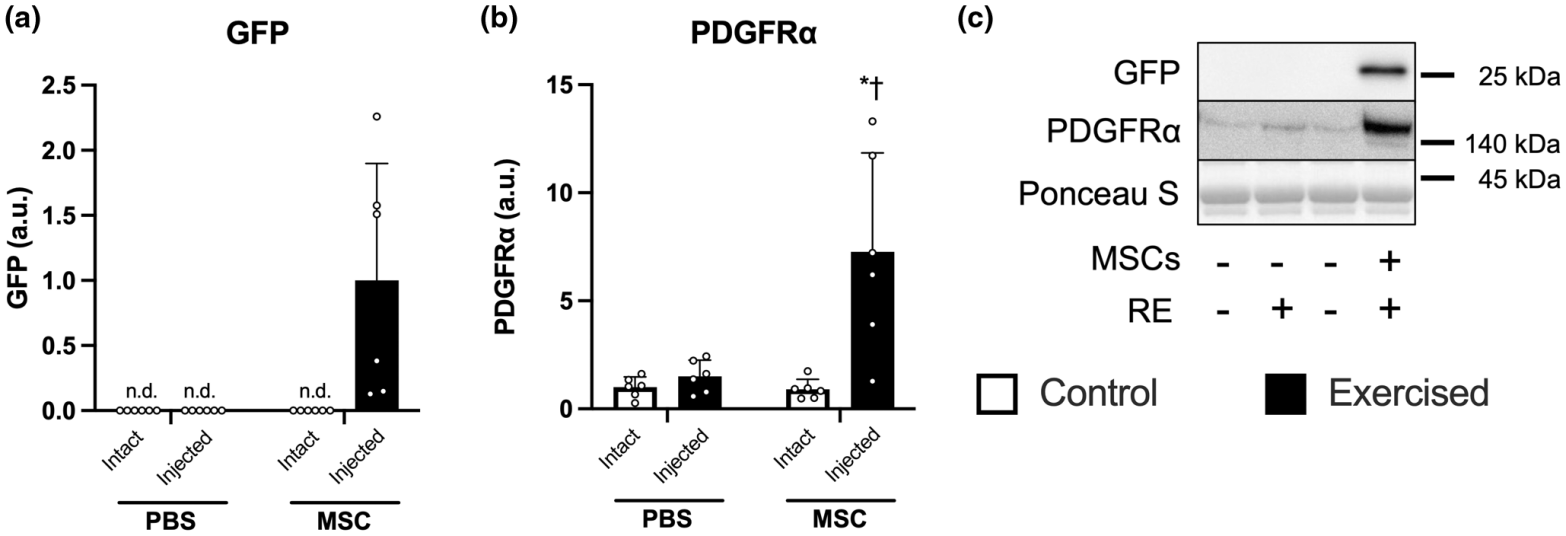
The expression of markers of mesenchymal stem cells (MSCs). The protein expression of GFP (a), PDGFRα (b), and representative bands (c). According to the group, exercise legs were injected with phosphate‐buffered saline (PBS)/MSCs, and control legs were kept intact, as indicated at the x‐axis. The data are expressed relative to the mean of the exercised leg in the MSC group (a) or the control leg in the PBS group (b) as the mean ± SD. *p < 0.05 versus control and ^†^ *p* < 0.05 versus the ipsilateral leg in the PBS group.

We detected GFP only in muscle injected with MSCs; the contralateral control leg showed no GFP expression (Figure 2a,c indicates representative blot data). The expression of PDGFRα, one of the surface markers of MSCs, increased only in an exercised leg injected with MSCs (PBS‐Exercise vs. MSC‐Exercise, *p* < 0.05; MSC‐Control vs. MSC‐Exercise, *p* < 0.05; Figure 2b).

### 3.2 Muscle protein synthesis and mTORC1 signaling

**FIGURE 3:**
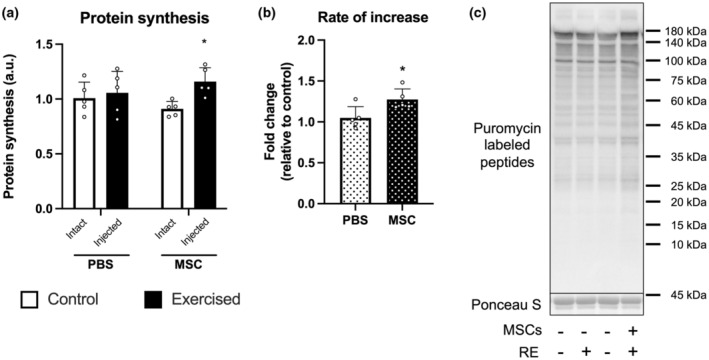
Muscle protein synthesis. Muscle protein synthesis was measured by the SUnSET method. (a) The rate of increase from the control leg (b) and representative bands (c). According to the group, exercise legs were injected with phosphate‐buffered saline (PBS)/mesenchymal stem cells (MSCs), and control legs were kept intact, as indicated at the x‐axis in the four‐bar graph. The data are expressed relative to the control leg in the PBS group (a) or PBS group (b) as the mean ± SD. *p < 0.05 versus control leg (a) or PBS group (b).

**FIGURE 4:**
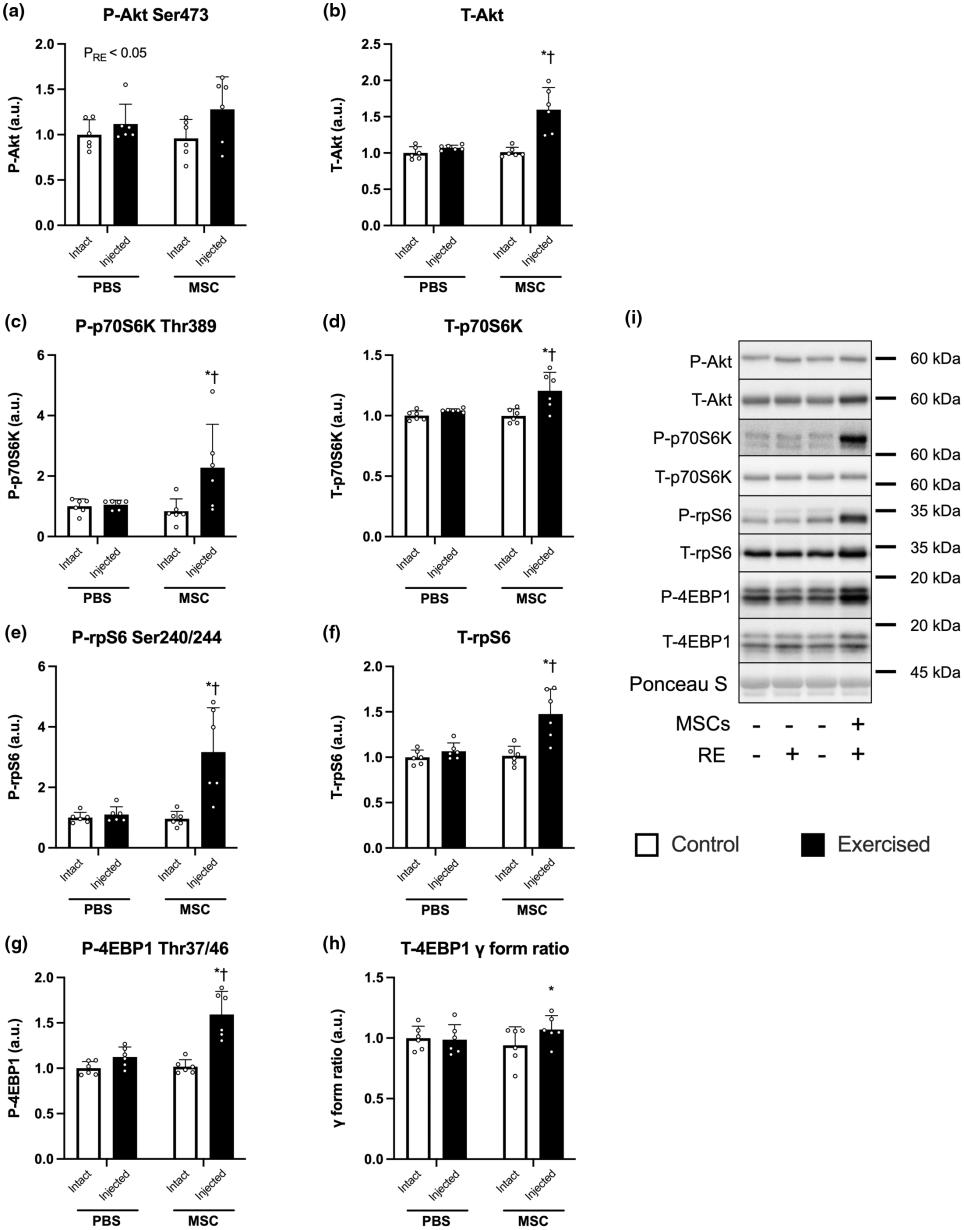
The expression of mTORC1 signal‐related factors. The protein expression of phosphorylated Akt (Ser473, a), Akt (b), phosphorylated p70S6K (Thr389, c), p70S6K (d), phosphorylated rpS6 (Ser240/244, e), rpS6 (f), phosphorylated 4EBP1 (Thr37/46, g), 4EBP1 gamma form ratio (h), and representative bands (i). According to the group, exercise legs were injected with phosphate‐buffered saline (PBS)/mesenchymal stem cells (MSCs), and control legs were kept intact, as indicated at the x‐axis. The data are expressed relative to the control leg in the PBS group as the mean ± SD. *p < 0.05 versus control leg and ^†^ *p* < 0.05 versus ipsilateral leg in the PBS group.

Muscle protein synthesis was activated in an exercised leg injected with MSCs (MSC‐Control vs. MSC‐Exercise, *p* < 0.05, Figure 3a,c indicates representative blot data), while no difference was observed in PBS‐Control and PBS‐Exercise. The ratio of muscle protein synthesis of control to an exercised leg in mice injected with MSCs was higher than that injected with PBS (*p* < 0.05, Figure 3b). The expression of phosphorylated Akt (Ser473) at basal state was increased by three bouts of resistance exercise (main effect of exercise, *p* < 0.05, Figure 4a,i indicates representative blot data). The expression of total Akt was increased only in exercised leg injected with MSCs (PBS‐Exercise vs. MSC‐Exercise, *p* < 0.05; MSC‐Control vs. MSC‐Exercise, *p* < 0.05; Figure 4b). The expression of phosphorylated (Thr389) and total p70S6K were increased only in exercised leg injected with MSCs (PBS‐Exercise vs. MSC‐Exercise, *p* < 0.05; MSC‐Control vs. MSC‐Exercise, *p* < 0.05; Figure 4c,d). Similar results were observed in the phosphorylated (Ser240/244) and total rpS6 (Figure 4e,f). The expression of phosphorylated 4EBP1 (Thr37/46) was increased only in exercised leg injected with MSCs (PBS‐Exercise vs. MSC‐Exercise, *p* < 0.05; MSC‐Control vs. MSC‐Exercise, *p* < 0.05; Figure 4g). Gamma form ratio of total 4EBP1 was increased in an exercised leg with MSCs (MSC‐Control vs. MSC‐Exercise, *p* < 0.05; Figure 4h).

### 3.3 Protein ubiquitination‐related factors

**FIGURE 5:**
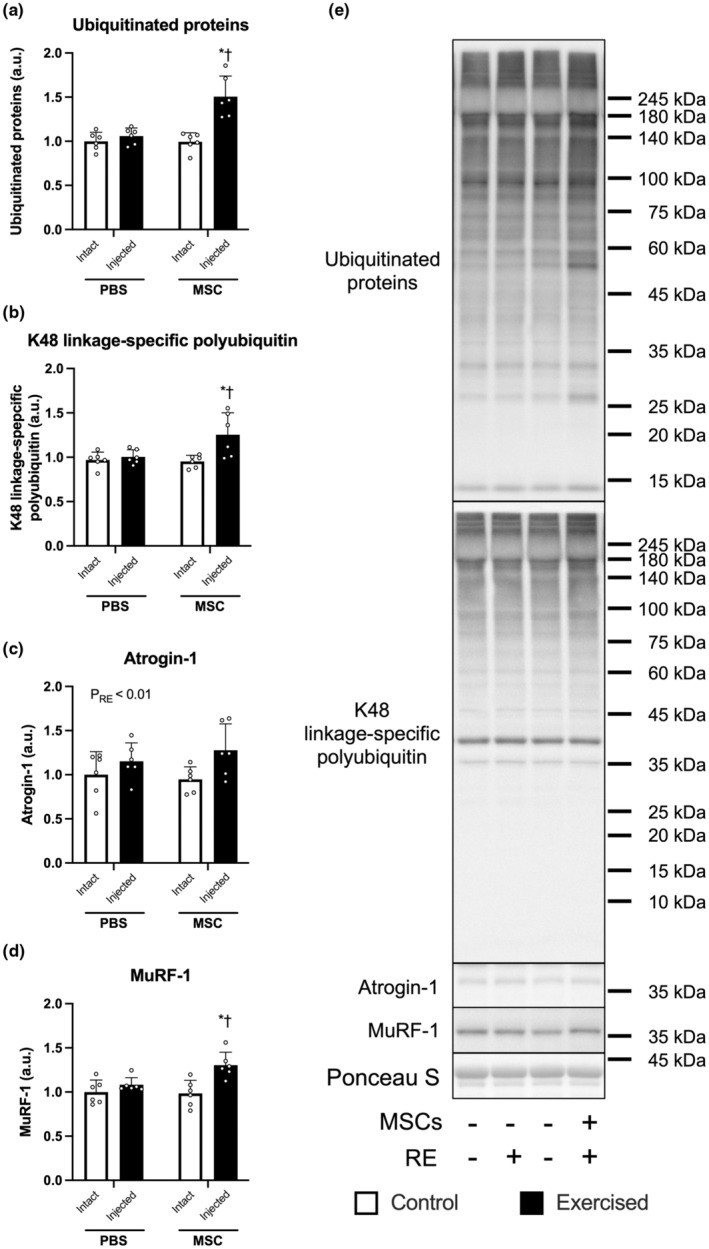
The expression of ubiquitin‐proteasome system‐related factors. The protein expression of ubiquitinated proteins (a), K48 linkage‐specific polyubiquitin (b), atrogin‐1 (c), MuRF‐1 (d), and representative bands (e). According to the group, exercise legs were injected with phosphate‐buffered saline (PBS)/mesenchymal stem cells (MSCs), and control legs were kept intact, as indicated at the x‐axis. The data are expressed relative to the control leg in the PBS group as the mean ± SD. *p < 0.05 versus control leg and ^†^ *p* < 0.05 versus ipsilateral leg in the PBS group.

The expression of ubiquitinated proteins (which was measured by western blotting) was increased only in the exercised leg injected with MSCs (PBS‐Exercise vs. MSC‐Exercise, *p* < 0.05; MSC‐Control vs. MSC‐Exercise, *p* < 0.05, Figure 5a,e indicates representative blot data). A similar result was observed in the expression of K48 linkage‐specific polyubiquitin (Figure 5b). The expression of atrogin‐1 was increased by three bouts of resistance exercise (main effect of exercise, *p* < 0.01, Figure 5c). On the other hand, the expression of MuRF‐1 was increased only in the exercised leg injected with MSCs (PBS‐Exercise vs. MSC‐Exercise, *p* < 0.05; MSC‐Control vs. MSC‐Exercise, *p* < 0.05; Figure 5d).

### 3.4 Autophagy‐related factors

**FIGURE 6:**
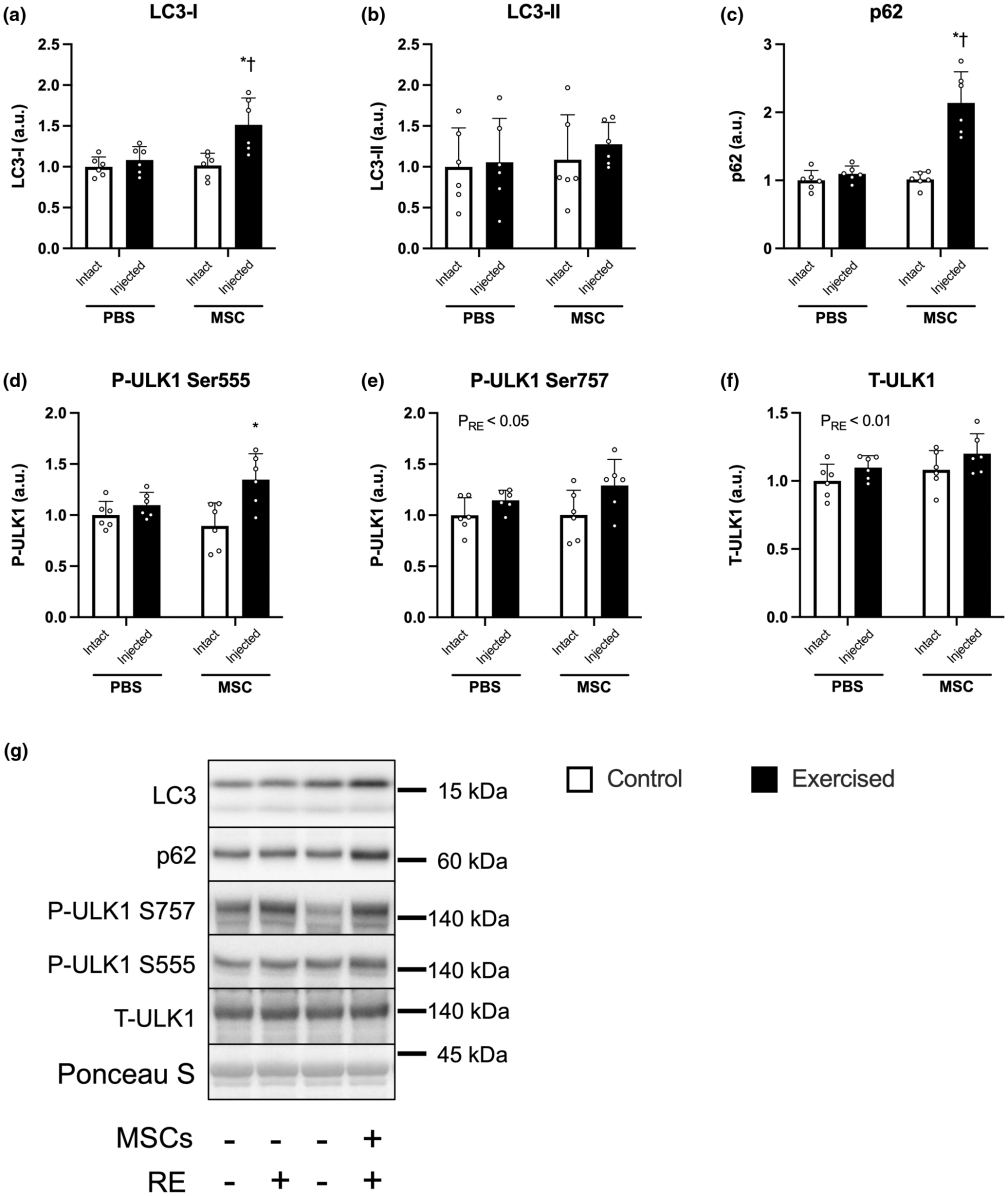
The expression of autophagy‐related factors. The protein expression of LC3‐I (a), LC3‐II (b), p62 (c), phosphorylated ULK1 (Ser555, d), phosphorylated ULK1 (Ser757, e), ULK1 (f), and representative bands (g). According to the group, exercise legs were injected with phosphate‐buffered saline (PBS)/mesenchymal stem cells (MSCs), and control legs were kept intact, as indicated at the x‐axis. The data are expressed relative to the control leg in the PBS group as the mean ± SD. *p < 0.05 versus control leg and ^†^ *p* < 0.05 versus the ipsilateral leg in the PBS group.

The expression of LC3‐I was increased only in the exercised leg injected with MSCs (PBS‐Exercise vs. MSC‐Exercise, *p* < 0.05; MSC‐Control vs. MSC‐Exercise, *p* < 0.05; Figure 6a). In contrast, three bouts of resistance exercise and the injection of MSCs did not change the expression of LC3‐II (Figure 6b). The expression of p62 was increased only in the exercised leg injected with MSCs (PBS‐Exercise vs. MSC‐Exercise, *p* < 0.05; MSC‐Control vs. MSC‐Exercise, *p* < 0.05; Figure 6c). Similar results were observed for the expression of phosphorylated ULK1 at Ser555 (Figure 6c). Three bouts of resistance exercise increased the expression of phosphorylated ULK1 at Ser757 and total ULK1. No significant effect of the injection of MSCs was observed (main effect of exercise, *p* < 0.05 and *p* < 0.01, respectively, Figure 6e,f).

### 3.5 Muscle satellite cell‐related factors

**FIGURE 7:**
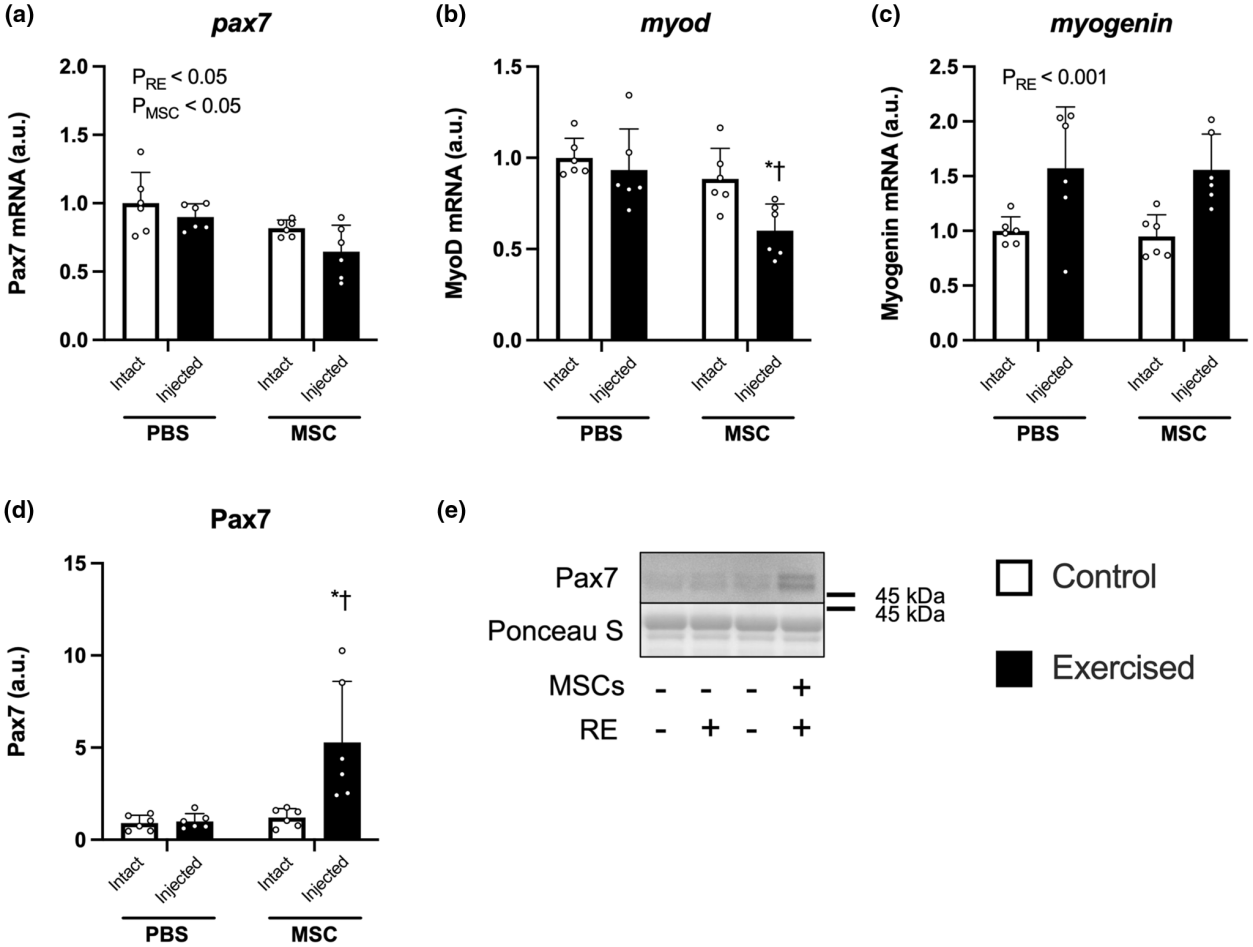
The mRNA expression of muscle satellite cell‐related markers. The expression of genes encoding Pax7 (a), MyoD (b), and myogenin (c). Protein expression of Pax7 (d) and the representative bands (e). According to the group, exercise legs were injected with phosphate‐buffered saline (PBS)/mesenchymal stem cells (MSCs), and control legs were kept intact, as indicated at the x‐axis. The data are expressed relative to the control leg in the PBS group as the mean ± SD. *p < 0.05 versus control and ^†^ *p* < 0.05 versus ipsilateral leg in the PBS group.

The mRNA expression of Pax7 was decreased by resistance exercise and decreased in both sides of the legs in mice injected with MSCs (main effect of exercise and MSCs, *p* < 0.05 and *p* < 0.001, respectively, Figure 7a). The mRNA expression of MyoD was decreased only in the exercised leg injected with MSCs (PBS‐Exercise vs. MSC‐Exercise, *p* < 0.05; MSC‐Control vs. MSC‐Exercise, *p* < 0.05; Figure 7b). The mRNA expression of myogenin was increased by three bouts of resistance exercise (main effect of exercise, *p* < 0.05, Figure 7c). The protein expression of Pax7 was increased only in the exercised leg injected with MSCs (PBS‐Exercise vs. MSC‐Exercise, *p* < 0.05; MSC‐Control vs. MSC‐Exercise, *p* < 0.05; Figure 7d,e).

### 3.6 IGF‐1 and IGFBPs

**FIGURE 8:**
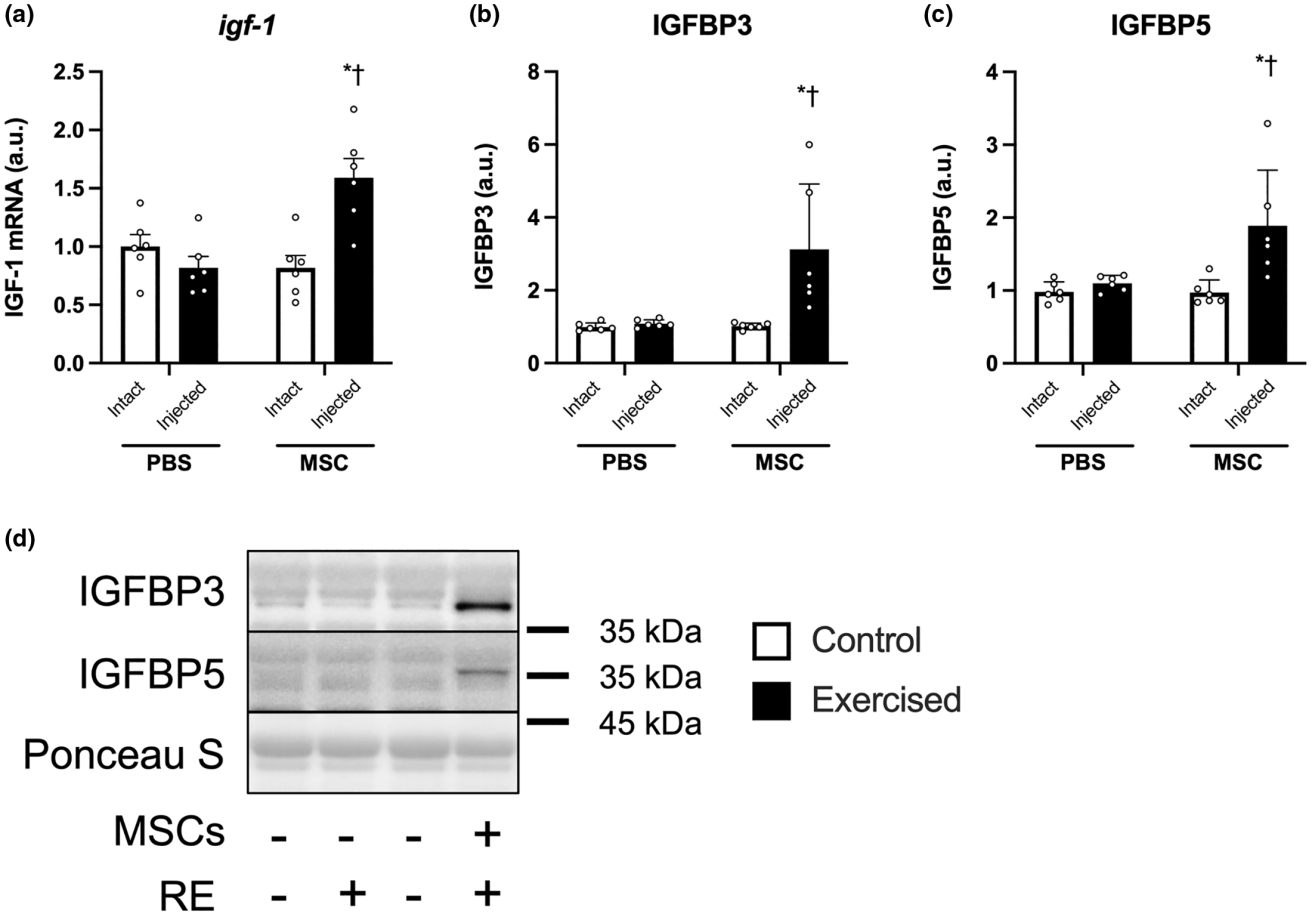
The expression of IGF‐1 and IGFBPs. The expression of a gene encoding IGF‐1 (a), protein expression of IGFBP3 (b), IGFBP5 (c), and representative bands (d). According to the group, exercise legs were injected with phosphate‐buffered saline (PBS)/mesenchymal stem cells (MSCs), and control legs were kept intact, as indicated at the x‐axis. The data are expressed relative to the control leg in the PBS group as the mean ± SD. *p < 0.05 versus control and ^†^ *p* < 0.05 versus ipsilateral leg in the PBS group.

The mRNA expression of IGF‐1 was increased only in the exercised leg injected with MSCs (PBS‐Exercise vs. MSC‐Exercise, *p* < 0.05; MSC‐Control vs. MSC‐Exercise, *p* < 0.05; Figure 8a). To clarify whether the injection of MSCs affects availability of IGF‐1, we also determined the expression of IGFBPs which inhibits IGF‐1 to the receptor and prolong the half‐life of IGF‐1. Similar results were observed in the protein expression of IGFBP3 and IGFBP5 (Figure 8b,c).

## 4 DISCUSSION

We examined the effect of intramuscular injection of MSCs on the muscle protein metabolism 72 h after the three successive bouts of resistance exercise. The main findings are as follows: (1) the injected MSCs remained in the muscle for a week during the entire resistance training period; (2) the injection of MSCs enhanced muscle protein synthesis and the activity of mTORC1 signaling at 72 h post‐exercise; (3) the injection of MSCs upregulated muscle protein breakdown systems, especially the ubiquitin‐proteasome system at 72 h post‐exercise; (4) the injection of MSCs increased the transcription of IGF‐1 and expression of IGFBPs at 72 h post‐exercise. These results suggest that intramuscular injection of MSCs increases muscle protein turnover at the basal state after repeated bouts of resistance exercise compared to control muscle.

The injected MSCs remained in the muscle for 7 days, indicated by the detection of GPP and the increased expression of PDGFRα. This observation aligns with our previous study (Takegaki et al., 2021). MSCs have an accumulative capacity to the inflammatory area (Eggenhofer et al., 2014). Resistance exercise acutely evokes local inflammatory responses in the exercised muscle (Buford et al., 2009; Townsend et al., 2016; Vella et al., 2011). Therefore, repeated bouts of resistance exercise might increase the retention of MSCs in the present study. Further studies are required to elucidate this point, as the mice injected with MSCs without exercise are not provided in this study. Our findings suggest that the injected MSCs remained in the muscle for a week, even after repeated bouts of resistance exercise.

In the exercised legs, muscle protein synthesis and mTORC1 signaling were not activated by the injection of PBS but by that of MSCs 72 h post‐exercise. After a single bout of resistance exercise, muscle protein synthesis increases at 3 h and remains elevated for 48 h (MacDougall et al., 1995; Phillips et al., 1997). In the rodent exercise model used in the present study, the elevation of muscle protein synthesis is confirmed until 24 h after the exercise bout (Ogasawara et al., 2016). However, 48 h after the 18 sessions of the resistance exercise bout, the activation of the mTORC1 signal is reported to return to the basal level (Takegaki et al., 2019). These findings suggest that the muscle protein synthesis returned to the basal level in the exercised leg injected with PBS, and the injection of MSCs kept muscle protein anabolism elevated. Our previous study reported that the intramuscular injection of the same number of cells (2.0 × 10^6^) activated muscle protein synthesis and mTORC1 signal for 7 days (Takegaki et al., 2021). Another study reported that injection of MSCs (1.0 × 10^6^, once per every week) increased phosphorylation of p70S6K in mdx mouse skeletal muscle four times (Pinheiro et al., 2012). These findings align with ours and suggest that intramuscular injection of MSCs augments basal muscle protein synthesis and mTORC1 signaling activation. The injection of MSCs may augment muscle hypertrophy with resistance training by increasing basal muscle protein synthesis during training. Future study is required to clarify the effect of MSC injection on muscle hypertrophy.

The injection of MSCs did not increase LC3‐II, an indicator of autophagosome formation, but increased ubiquitinated proteins and K48‐linkage‐specific polyubiquitin, which are to be degraded by 26S proteasomes (Bodine & Baehr, 2014). Interestingly, the injection of MSCs affected only one E3 ubiquitin ligase (MuRF‐1). The mechanism for this is unclear. Intramuscular injection of MSCs evokes upregulation of transcription of these two E3 ubiquitin ligases at 2 days post‐injection but not at 7 days post‐injection (Takegaki et al., 2021). Mascher et al. reported that the mRNA expression of atrogin‐1 was lower before and after the second resistance exercise session than before the first (Mascher et al., 2008). That finding suggests that repeated bouts of resistance exercise might have attenuated atrogin‐1 expression, resulting in no significant difference between the exercised legs of placebo and MSC groups. Elucidating the mechanisms for this would contribute to understanding the adaptation of proteolytic aspects in the combination of exercise and MSC injection. Our findings suggest that intramuscular injection of MSCs likely activates the protein degradation system by upregulating MuRF‐1.

In the present study, the injection of MSCs did not increase the transcription of myogenic factors. In our previous study, the increase in myogenin mRNA expression was observed at 2 days post‐injection but not at 7 days (Takegaki et al., 2021), suggesting that the time in the present study (1 week after the intramuscular injection of MSCs) was too late to observe the effect of MSCs on transcription of myogenic factors, even with the exercise stimulus. In contrast, a previous study reported that the combination of intramuscular injection of MSCs and downhill running increased Pax7‐positive nuclei at 1 ‐week post‐injection in mouse skeletal muscle (Zou et al., 2015). We did not perform morphological analysis but confirmed that the injection of MSCs and bouts of resistance exercise increased Pax7 protein expression in the present study. On the other hand, the transcription of Pax7 was not increased simultaneously. In our previous study, the mRNA expression of Pax7 was not increased at 2 and 7 days after the injection of MSCs, despite the mRNA expressions of myogenin and MyoD increased (Takegaki et al., 2021). These facts suggest that the transcription of Pax7 may be increased at quite an acute phase after the injection of MSCs. Overall, the current study suggests that bouts of resistance exercise and MSC injection might positively affect the transcription of myogenic factors in the early phase (e.g., 2 days) after the injection.

In line with a previous study, the injection of MSCs increased the transcription of IGF‐I (Takegaki et al., 2021), which might contribute to the activation of muscle protein synthesis in the muscle injected with MSCs. Moreover, the injection of MSCs also increased the expressions of IGFBP3 and IGFBP5. IGFBPs, which modulate IGF‐I activity, are secreted by various cells and prolong the half‐life of IGFs (Duan & Xu, 2005; Firth & Baxter, 2002). In skeletal muscle, IGFBP3 and IGFBP5 regrow muscles from the atrophied condition in rats (Spangenburg et al., 2003). IGFBP3 and IGFBP5 increase myoblast differentiation in vitro (Foulstone et al., 2003; James et al., 1993). These findings suggest that the injected MSCs differentiated into skeletal muscle cells and integrated into the resident muscle fibers. However, other studies reported that overexpression of IGFBP5 interfered with skeletal muscle development and differentiation in vivo and in vitro (James et al., 1996; Salih et al., 2004). Whether the elevation of IGFBPs in the present study was optimal for promoting the differentiation of MSCs into skeletal muscle is unclear and should be clarified in future studies.

The present study has some limitations. First, we did not evaluate the site‐specific effects of the injection of MSCs. Intramuscularly injected MSCs primarily affect the injection site in skeletal muscle (Takegaki et al., 2021). Also, a previous study showed that the intracardiac injection of adult stem cells on the post‐ischemic injury site showed a therapeutic effect but has reported that this was due to an acute immune response (Vagnozzi et al., 2020). After the skeletal muscle damage, accumulation of macrophages, activation of satellite cells, and paracrine of IGF‐1 are observed (Tonkin et al., 2015). Some of these observations overlap the results obtained in the present study, and we previously confirmed infiltration of mononuclear cells at the injected site after intramuscular injection of MSCs (Takegaki et al., 2021), suggesting that the immune response, muscle damage, and regeneration process might have caused the observed results. The detailed histological analysis (e.g., immunofluorescence staining for the factors involved in muscle protein anabolism/catabolism and macrophages) would contribute to a better understanding of the effect of intramuscular injection of MSCs. Second, we did not evaluate the muscle protein synthesis throughout the intervention period. The evaluation would show the full potential of MSCs by providing a comprehensive picture of the impact of intramuscular injection of MSCs on resistance training, including effects on the basal state and the acute response after exercise. Third, we did not provide a group that received only MSC injections without exercise. By providing this group, we can investigate whether the changes observed in the present study are solely due to the injections or the synergistic effect of the combination with exercise.

In conclusion, intramuscular injection of MSCs activated transcription of IGF‐1 and muscle protein synthesis 72 h after the three bouts of resistance exercise. The injection of MSCs also increased protein ubiquitination, suggesting augmented basal muscle protein turnover after consecutive resistance exercise. Although more detailed examinations of muscle protein turnover and long‐term effects are required, these findings provide a foundation for the application of MSCs and the possibility that the injection of MSCs can facilitate muscle hypertrophy in resistance training.

## AUTHOR CONTRIBUTIONS

Conception and design of the work: J.T., K.S., Y.K., T.F., S.K., and S.F. Acquisition and analysis: J.T. and K.S. Interpretation of Data: J.T. and S.F. Writing the original draft: J.T. Review and editing: K.S., Y.K., T.F., K.S., and S.F. All authors approved the final version.

## FUNDING INFORMATION

The Ritsumeikan Global Innovation Research Organization project at Ritsumeikan University supported this work.

## CONFLICT OF INTEREST STATEMENT

The authors declare no competing interests.

## ETHICS STATEMENT

All experiments were approved by the Ethics Committee for Animal Experiments at Ritsumeikan University (BKC‐2018‐046) and performed in accordance with the guidelines of the committee.

## Supporting information


Figure S1.

Table S1.

## Data Availability

The data that support the findings of this study are available from the corresponding author upon reasonable request.
