# Supplementary figures and images for: Intramuscular injection of mesenchymal stem cells augments basal muscle protein synthesis after bouts of resistance exercise in male mice
Source: Physiol Rep. 2024 Apr 11;12(7):e15991. doi: 10.14814/phy2.15991 (PMC11009371; doi:10.14814/phy2.15991)

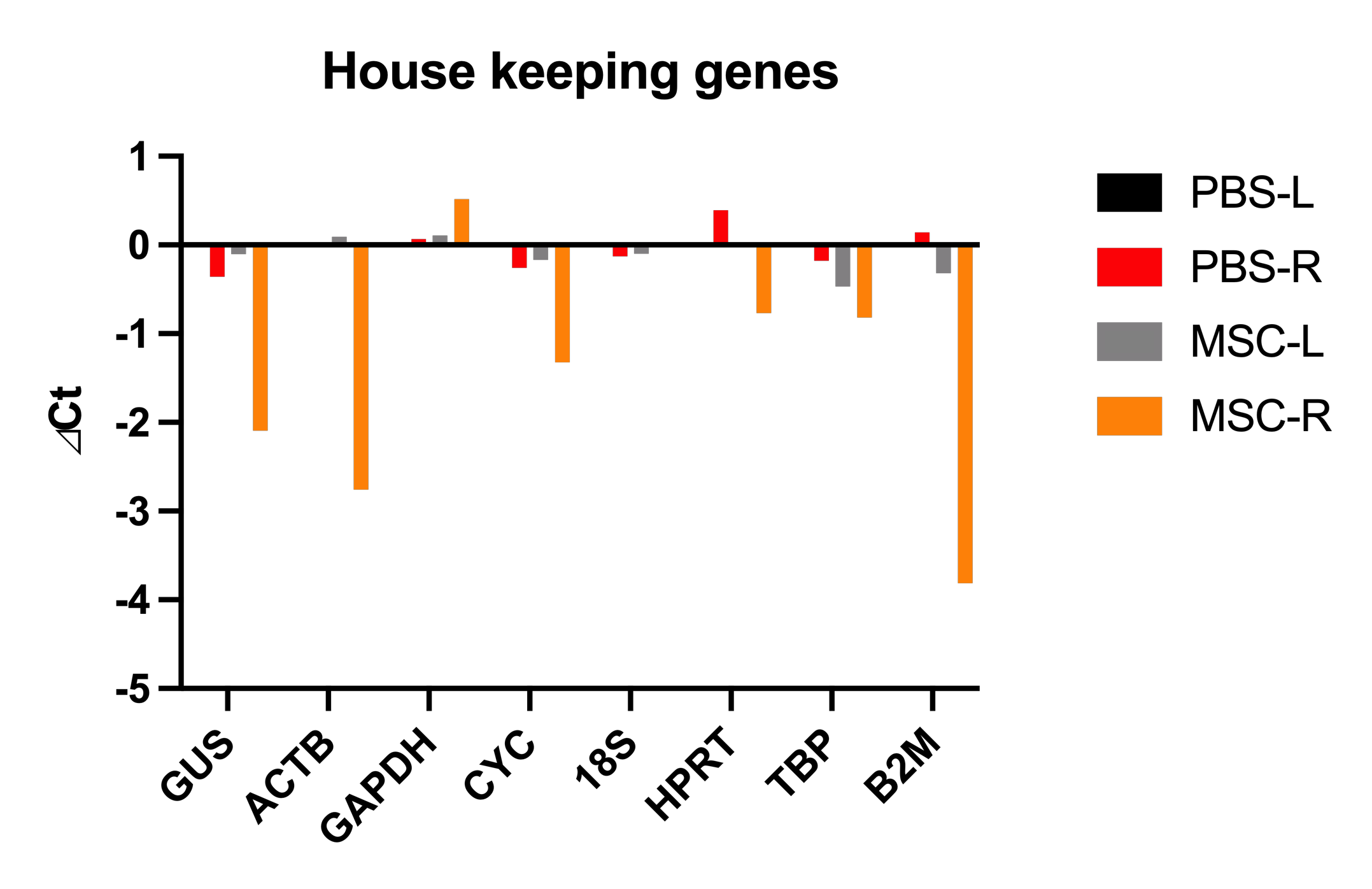

Supplement: Supplementary file 1 — Figure S1. [file PHY2-12-e15991-s002.tif]
