# Supplementary material for: Intramuscular injection of mesenchymal stem cells augments basal muscle protein synthesis after bouts of resistance exercise in male mice
Source: Physiol Rep. 2024 Apr 11;12(7):e15991. doi: 10.14814/phy2.15991 (PMC11009371; doi:10.14814/phy2.15991)
Supplement: Supplementary file 2 — Table S1. [file PHY2-12-e15991-s001.docx]

Table 1S. Primer sequences for qPCR of housekeeping genes.

| Gene | Forward primer (5’-3’) | Reverse primer (5’-3’) |
| --- | --- | --- |
| *Gus* | CACGGCGATGGACCCAAGAT | CCCATTCACCCACACAACTGC |
| *Actb* | GGCTGTATTCCCCTCCATCG | CCAGTTGGTAACAATGCCATGT |
| *Gapdh* | CTCCCACTCTTCCACCTTCG | GCCTCTCTTGCTCAGTGTCC |
| *Cyc* | TCCGACTGTGGACAGCTCTA | ATTGCGAGCAGATGGGGTAG |
| *18s* | CCTGGATACCGCAGCTAGGA | GCGGCGCAATACGAATGCCCC |
| *Hprt* | GCTTGCTGGTGAAAAGGACCTCTCGAAG | CCCTGAAGTACTCATTATAGTCAAGGGCAT |
| *Tbp* | TATGACCCCTATCACTCCTG | TTCTTCACTCTTGGCTCCTGT |
| *B2m* | TTCTGGTGCTTGTCTCACTGA | CAGTATGTTCGGCTTCCCATTC |
